# Palliative Care for the Elderly With Heart Diseases in Tertiary Health care: A Concept Analysis

**DOI:** 10.1177/10499091231213606

**Published:** 2023-11-14

**Authors:** Sana Ali, Jane Tyerman

**Affiliations:** 1 School of Nursing, Faculty of Health Sciences, 6363 The University of Ottawa, Ottawa, ON, Canada

**Keywords:** palliative care, elderly, aged, geriatrics, tertiary healthcare, inpatients, heart diseases, heart failure, cardiac

## Abstract

**Background:**

The increasing incidence of heart failure (HF) in the elderly leads to increased mortality, hospitalization, length of hospital stay, and health care costs. Older adults often face multiple drug treatments, comorbidities, frailty, and cognitive problems, which require early palliative care. However, these patients do not receive adequate palliative care.

**Objective:**

This concept analysis aimed to develop an in-depth understanding of palliative care for elderly patients with cardiac diseases in tertiary care.

**Design:**

The analysis was guided by Walker and Avant's method, and databases were searched using keywords, such as palliative care, tertiary care, elderly, and heart. Covidence was used to review the results using the inclusion and exclusion criteria.

**Results:**

The World Health Organisation’s definition of palliative care is widely accepted. Palliative care for older adults with heart disease in tertiary care is preceded by chronic illness, polypharmacy, symptom burden, physical and cognitive decline, comorbidities, and psychosocial/spiritual issues. The main attributes of palliative care for this population include health care professionals and patient education, holistic patient/family-centered care, symptom management, shared decision-making, early integration, advanced care planning, and a multidisciplinary approach. Palliative care improves elderly cardiac patients' and their family satisfaction while reducing readmission, hospital stays, and unnecessary invasive procedures.

**Conclusion:**

Collaboration between hospitals, community organizations, transitional palliative care services, and research has the potential to improve early palliative care and the well-being of the elderly cardiac population. Advanced Practice Nurses (APNs) competencies play a crucial role in promoting palliative care in the elderly HF population.

## Introduction

Cardiovascular disease remains the leading cause of death worldwide and was responsible for an estimated 17.9 million deaths in 2019.^1^ Despite optimal cardiac treatment, many cardiovascular diseases can progress to heart failure (HF), a debilitating syndrome characterized by a significant symptom burden and reduced quality of life.^2^ It is important to note that hospitalizations related to heart failure contribute to an estimated US$108 billion in annual expenses globally.^3^ Moreover, there has been a notable increase in the prevalence of heart failure in elderly patients, owing to the increasing aging population.^4^ The complexity of heart failure management in geriatric patients is further compounded by comorbidity, frailty, physical and cognitive decline, and the need for multiple medications (polytherapy). Cognitive dysfunction has been reported in a substantial proportion of patients with heart failure, ranging from 17% to 35%, and is associated with worse heart failure outcomes in hospitals, including increased length of hospital stay, higher readmission rates, and increased mortality.^3^

As HF is a chronic condition with high morbidity and mortality rates, palliative care (PC) can assist in managing symptoms to optimize the quality of life. According to the World Health Organization, approximately 38.5% of the 40 million people worldwide who require PC annually are cardiac patients.^5^ Most academic teaching hospitals integrate palliative care into their practice, research, and education. PC provision in hospitals reduces health care spending by decreasing the length of stay and number of procedures and interventions performed near the end of life.^6^ Despite this, many studies have concluded that the rate of PC referrals in hospitals is low and that PC often integrated late into the illness.^7-8^ A study in 74 hospitals showed that PC consultations were relatively few for older patients with HF and were only provided during the last few hours of a patient’s life.^9^ Another 15-year national study in the USA found that only 4.5% of patients admitted with myocardial infarction-related cardiogenic shock in hospitals received PC, most of whom had a higher number of comorbidities, a higher incidence of acute organ failure, and were above 80 years.^10^ Barriers to providing early PC to elderly cardiac patients include conflicting philosophies, misconceptions, lack of knowledge of PC among nurses and other health care professionals (HCP), and insufficient communication with patients and their families.^11,12-13^

## Aim

This concept analysis aims to develop an in-depth understanding of palliative care for elderly patients with heart disease in a tertiary health care setting.

## Method

### Study Design

The concept analysis was guided by Walker and Avant’s approach. This involves eight steps: selecting the concept, outlining its purpose, determining relevant uses, describing attributes, examining a model case and other cases, identifying antecedents and consequences, and establishing key empirical referents.^14^ In the introduction section, the rationale for selecting the palliative care concept for elderly patients with heart disease in tertiary care is discussed in the context of the current literature, and the purpose of the analysis is also mentioned. In the results section, the use of the concept, antecedent, attributes, and consequences of the concept are discussed, along with the model, borderline and contrary cases, and empirical referents.

### Search Strategy and Data Sources

In a preliminary search using Google Scholar, keywords and concepts related to PC in elderly patients with cardiac diseases were identified. The search terms are listed in Table 1. The following databases were searched: CINAHL, MEDLINE, PsycINFO, Scopus, Age Line, PubMed, and Google Scholar. Keywords suggested terms, and truncations specific to the database were used in subject headings. Each key concept (palliative care, elderly, cardiac diseases, and tertiary care) was searched separately using ‘OR’ between the literature search terms. These results were combined using ‘AND’ to retrieve the articles related to the concept (Table 1). This strategy improves the number and relevance of the search results. The titles and abstracts were further screened using the inclusion and exclusion criteria. The inclusion criteria were as follows: English language, full-text availability, peer-reviewed literature published between 2011 and 2021, and a key population focus of age ≥65 years. The exclusion criteria were as follows: study results related to multiple diseases other than HF, and populations below the age of 65 years.

**Table 1.** Search Strategy.

**P (Population)**	**I (Intervention)**	**C (Context)**	**O** **(Outcome)**
Elderly **OR** age* **OR** old* **OR **geriatric*	Palliative care **OR **Palliative **OR** palliative*	Heart **OR** cardiac **OR** cardiovascular **OR** cardi*	N/A
		Tertiary **OR** acute care **OR** hospital* **OR** inpatient*	

Note: Key terms in PICO format were used in each database. Schiavenato M, Chu F. PICO: What it is and what it is not. Nurse Education in Practice. 2021; 56:103194. https://doi.org/10.1016/j.nepr.2021.103194.

### Data Management and Analysis

**Figure 1.**
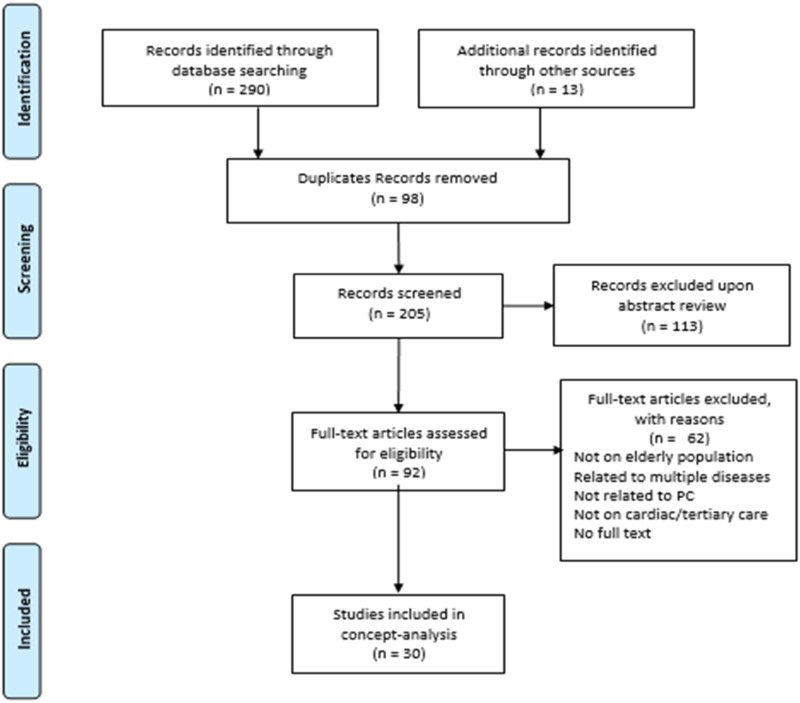
PRISMA diagram for data management and analysis.

A total of 303 articles were imported into the Covidence Database.^15^ Ninety-eight duplicates were removed, and abstracts were screened for relevance. The remaining 92 full-text articles were assessed for their eligibility. Studies were excluded owing to outcomes related to multiple diseases, the absence of key concepts (Table 1), and the unavailability of full-text articles. Thirty articles published between 2012 and 2021, primarily from North America, Europe, Asia, and Australia were reviewed (Figure 1).

## Results

Table 2 summarizes the 30 articles included in the analysis. All details of the studies, including author, year, type of study, origin, focus of study, antecedents, attributes, and consequences, are presented in the table. The results of the analysis highlight the definition of palliative care, use of the PC concept in literature in the context of older adults with heart disease, antecedents, attributes and consequences, model, borderline and contrary cases, and empirical referents.

**Table 2.** Summary of Included Studies (n = 30).

Author, Year Country/Study	Focus of the article	Antecedent	Attributes	Consequences
Abete et al, 2013^16^, Italy, Review	Current practice and problems related to elderly heart failure (HF) patients	Chronic disease process, cognitive dysfunction, frailty, comorbidities, symptom burden polypharmacy, nonadherence to therapy	Symptom management (pain, depression, sleep), ACP, patient/caregiver education, device implantation decision, multidisciplinary team	Improve self-care and patient participation, family/caregiver support, and social and emotional support for patients
Bader et al, 2017^17^, USA, Europe, and the middle east gulf region Review	Discuss issues related to diagnostic challenges, disease burden, medication, epidemiology, and palliative care (PC) impact on the elderly population	Comorbidities, polypharmacy, non-adherence, frailty, physical/functional and cognitive decline	Early PC, holistic care, symptom management, care coordination, multidisciplinary team approach, shared decision-making, and family bereavement support	Improve self-care, highest quality of life, maximize patient comfort, physical and psychological well-being of the family, and avoid futile and uncomfortable interventions at the end-of-life (EOL)
Bakitas et al, 2013^8^, USA, Retrospective study	Characterize the HF population receiving palliative care consultations (PCCs)	HF is an unpredictable disease with high mortality, symptom burden, physical and psychological needs	Early PC, ACP, symptom management, goal of care (GOC)	Improved quality of life (QOL) and reduced symptom intensity, reduced readmission
Butler et al, 2015^18^, USA, Retrospective study	Assess the frequency and correlates of documented advance directives (ADs) among patients hospitalized for heart failure (HF)	HF is an unpredictable disease with high mortality, sudden death, physical, psychosocial, and spiritual issues	Identify treatment goals, AD documentation, health care team communication, multidisciplinary approach	Provide support to the patient, ACP in HF lowers health care costs and lowers the risk of in-hospital death
Chan et al,2016^19^, China, Correlation descriptive study	Examine the quality of life and PC needs of elderly patients with HF	Spiritual needs, symptom burden, symptoms affecting physical, psychological, and social functioning and QOL	Symptom control, spiritual and psychological care	Improving the QOL
Chester et al, 2021^7^, UK, Qualitative study	Explore views of patients and carers who participated in the integrated pilot service (cardiology and PC)	Frailty, uncertainty related to disease trajectory, multimorbidity	Early referral to PC, PC communication with patient and caregiver, symptom control, PC education, shared decision making, family support, ACP communication, multidisciplinary approach, psychological and spiritual care	Early PC is cost cost-effective approach, improves quality of life, reduces symptom burden and hospital admission, help family and caregiver to cope
Denvir et al, 2015^20^, UK, Review	Future care planning in PC for patients with advanced heart disease	HF poor prognosis, severe symptom, high mortality, comorbidities, risk of sudden death	Timely introduction of PC, holistic care for HF people, ACP, shared decision-making, good communication	Improve symptoms and QOL, ACP in PC reduces hospital admissions, and health care resource utilization and improves patient satisfaction
Dionne-odom et al, 2014^21^, USA, Qualitative study	Translating and testing the ENABLE: CHF-PC concurrent PC model for older adults with heart failure and their family caregivers	HF is a chronic disease with high morbidity and fatality, symptom burden, functional limitation related to symptoms of physical and emotional suffering, and multiple hospitalizations	Early PC timely PC (at time of diagnosis), symptom management, selfcare, communication, care coordination, ACP, decision making, physical, psychological, emotional wellbeing, education for clinicians and patients, telemonitoring	Reduce physical and emotional stress, improve quality of life, patient, and caregiver satisfaction related to nurse coach
Ezekowitz et al, 2017^22^, Canada, Review	Canadian cardiovascular society guidelines for the management of heart failure	Polypharmacy, recurrent hospitalization, multimorbidity, frailty, symptom burden (physical psychosocial)	Patient/family-centered care, psychosocial, and spiritual support symptom management, GOC, ACP, multidisciplinary team, care coordination, self-care, telemonitoring, deprescribing, device deactivation, patient/caregiver education	Improve quality of life, better health outcomes, reduce cost, symptom burden, and health system utilization
Fernandes pedro and Reis-pina, 2021^9^, Spain. Cross-sectional study	Determine the prevalence of advanced heart failure in admitted patients, describe their management, and analyze the factors that influence their referral to specialized PC.	Comorbidities, functional decline, persistent severe symptom burden	Multidisciplinary approach, symptom management, physician training, spiritual support	Improve symptoms and quality of life, patient, and family satisfaction
Gerlich et al 2012^23^, Germany, Qualitative study	Explore older patients need for improving communication with patients with advanced HF.	Severe physical und psychosocial symptoms such as fatigue, pain, breathlessness, fear, and social isolation	Effective communication, goal setting, advanced care planning, holistic care	Relieve patient fears
Heidenreich et al, 2022^24^, USA, Literature review	American College of Cardiology/American heart association joint Committee on clinical practice guidelines for the management of heart failure	High morbidity and mortality, high readmission, frailty, polypharmacy, uncontrolled symptoms multimorbidity, and cognitive decline	Clarifying GOC, high-quality communication, shared decision-making, symptom management, interdisciplinary PC, caregiver support, ACP, and ICD, LVAD decisions	Greater benefits in QOL, anxiety, depression, and spiritual well-being compared with usual care
Huitema et al, 2018^25^, Canada, Quality improvement	Examines the concept of chronic disease management, the effect of HF management programs, and a systems approach to HF	Unpredictable illness trajectory and prognosis	Symptom management, facilitation of advance care planning, optimizing transitions, and EOL	Improvement in QOL, symptom management and reduction in use of medical services, hospital visits, admissions, and length of stay
Iyngkaran, 2018^26^, Australia, Feature article	Thematic series on congestive heart failure (CHF) aims to outline important gaps in guidelines for patients with multiple comorbidities and the elderly	Comorbidities, frailty, psychological, and social vulnerability as well as geriatric syndromes such as falls, confusion, uncertainty to diagnosis, and decline of independent function, self-care, and QOL with increased dependency, high readmissions rate	Early and appropriate PC, multidisciplinary care minimizing polypharmacy, consultation with families, specialist, general practitioners, and allied health teams	Reduce readmission rates
Kim & hwang, 2014^12^, South Korea, cross-sectional descriptive design	Investigate nurses’ PC knowledge, attitudes toward care of the dying, coping with death, and preparedness to PC practice for those with HF, and evaluate factors influencing preparedness to PC practice	Unpredictable disease trajectory physical and psychological burden onto patients and their families	Early integration, improve HCP knowledge and education	Provide care needs of patient with HF, assist them with ACP, symptom management, deal with psychological and social issues, and education improve attitudes and identify GOC along the trajectory of HF illness
LeMond et al, 2015^27^, USA, Review	Update care providers on the most recent primary literature published on decision making and PC in advanced HF.	Comorbidities including frailty, cognitive dysfunction, and depression, cognitive impairment	Symptom management and support of the patient and family, holistic approach, shared decision making, advance care planning	Improve the quality of life of patients, relieving physical, psychosocial, and spiritual pain and facilitate a good death
McIlyennan & Allen, 2016^6^, USA, Review	Summarize the evidence for PC in patients with heart failure, barriers to its integration, and future directions	Uncertainty in the disease trajectory, lack of knowledge, siloed care, HCP and patients’ communication failure, comorbidity and frailty, life saving devices with complex trade-offs, and a limited evidence base, high morbidity, and hospital admission	Early in the course of illness, multidisciplinary approach, shared decision making, and ACP, HCP education, symptoms relief, device deactivation psychological and spiritual support, families (bereavement) support, effective communication	Optimize QOL of patient and caregiver and ease the transition to the end of life. Reduce health care spending, length of stay and number of procedures and interventions performed near the end of life
Moretti et al, 2017^28^, Europe, Prospective, multicentre study	Assess the utility of the gold standards framework (GSF) in identifying risk of death and wider multidimensional needs in patients presenting to hospital with acute coronary syndrome	Multiple co-morbidity, frailty, complex multidimensional needs high risk of death or re-hospitalisation	PC tool (GSF) aim to identify people with complex needs requires supportive/palliative care and ACP. It assesses unintentional recent weight loss, performance status impairment, general physical decline, multiple hospitalisations during the last year and clinical worsening despite optimised medical therapy	Less likely to undergo coronary angiography and associated interventional procedures, raised awareness of end-of-life issues
Okumura et al 2018^4^, Japan, Review	Palliative and end-of-life care for heart failure aging population	High risk of sudden cardiac death, deterioration of QOL, heavy economic, social functional burden, physical and mental decline, frequent hospital admission, psychosocial and spiritual problems	Device deactivation, multi-disciplinary teams, support the patient, aid in future planning, early identification for PC, symptomatic management device implantation/deactivation, goals discussion with patients and caregivers	Improve QOL, minimize suffering in terms of physical pain and mental, social, and spiritual distress
Pere, 2012^29^, Canada, Review	Identify evidence-based strategies and frameworks that would aid nurses and other health care professionals (HCP) in the integration of PC into the care path of CHF patients	Unpredictable illness trajectory, lack of PC knowledge among acute care nurses, and lack of communication with patients and their families. Sudden death, psychosocial and spiritual needs of the patient and family	Early PC, patients and family’s education, symptom control, ACP, decision making on treatment preferences holistic fashion, device management, HCP, shared decision-making family-centred care, multidisciplinary education	Improvement of symptoms, patient function, and QOL. Enhances evaluation and treatment on physical, psychosocial, and spiritual levels. Enhances patient care, increases patient satisfaction, facilitates understanding of the diagnosis and prognosis, improves symptom control and decreases health care costs
Piers et al, 2021^30^, Belgium, Prospective multicentre study	Validate supportive and palliative care indicators tool (SPICT) as a prognostic tool for the risk of dying within 1 year in older hospitalized patients	Older adults deprived of PC due to prognostic uncertainty	SPICT may be used to identify older hospitalised patients at risk of dying within 1 year and who may benefit from a PC approach, early integration patient-centred communication, and ACP	PC approach including ACP leads to better quality of life for patients and less mental stress in family member
Schnell-hoehn et al, 2017^13^, Canada, Descriptive survey design	Explore the palliative care knowledge of cardiac nurses using the palliative care Quiz for nursing (PCQN)	Functional declines, need for cardiac nurses to be knowledgeable	Relief of suffering using early detection, GOC	Optimizing quality of life and palliation
Shah et al, 2013^22^, USA, Review	Update on the role of PC in HF patients, including the current state of PC involvement in HF, the potential impact on QOL, PC models, and symptom management	High risk of sudden cardiac death, unmet social and spiritual needs, underutilized PC consultation	Early patients and families’ communication symptom management, coordinated care, ACP, device management multidisciplinary approach	Improve patients’ satisfaction. Fewer invasive procedures and interventions at the EOL, decreased length of stay, and shorter admissions to intensive care units
Sidebottom et al, 2015^31^, USA, Randomized trial	Assess if inpatient PC for HF patients is associated with improvements in symptom burden, depression, QOL, or differential use of services	High symptom burden, reduced QOL high mortality functional impairment	Supports functional status, GOC, communication, complex decision making	Improvement in symptom control, QOL, and increase completion of ACP depressive symptoms
Sobanski et al, 2020^2^, Poland, Review (guidelines)	Discuss PC for people living with cardiac disease	HF is progressive syndrome with significant distress and limiting QOL, fragmented medical care, polypharmacy multimorbidity, cognitive disorders, lower mood, worsened functional status, and lack of social support, increased hospitalizations	Multi-professional team, needs-based PC model, symptom control (ESAS), decision-making support, ACP, social support, open communication, spiritual care, holistic approach, bereavement counseling, interdisciplinary approach, HCP training, device management, deprescribing, NAT: PD-HF is validated tool evaluate the PC needs of the patient	Relieve symptoms and improve quality of life, reduce suffering
Teixeira et al, 2016^32^, Europe, Review	Management of acute heart failure in elderly patients	Unplanned hospital admissions, multiorgan failure, co-morbidities, sedentary lifestyle, disability, frailty and increased morbidity, mortality, readmission rates	PC at an early stage, avoid unnecessary and harmful diagnostics and treatments, symptomatic treatment	Improve patient and family satisfaction, decrease health care utilization
Tersalvi et al, 2021^3^, Europe, Review	Offer an insight into current literature and provide a clinically oriented, patient-tailored approach regarding assessment, treatment and follow-up of elderly patients admitted for HF.	Polypharmacy, increased morbidity, disability, frailty, longer recovery time, comorbidities, higher readmission rates, health care cost and mortality, limited option for mechanical circulatory support and cardiac transplantation, cognitive dysfunction	Early PC, deprescribing, patient-tailored approach, symptom control, device deactivation, treatment specific to goals, psychological support, multimodal approach, multidisciplinary cardiac rehabilitation program, caregiver QOL, tailored telemedicine, outpatient follow-ups, involve the patient in treatment decisions	Avoid unnecessary diagnostics and treatments, improve QOL, reduce in-hospital mortality, and readmission rates, increase patient and family satisfaction
Turris et al, 2021^11^, USA, pretest, post-test pilot study	Determine if providing nurses with education on the timing and content of PC conversations would improve their perceived skill and knowledge	Unpredictable disease trajectory, delayed PC services, unmet physical, emotional, spiritual, and social needs, lack of knowledge about the timing and content of PC conversations	Symptom management and improved QOL, timely PC, holistic care, patient and family-centered approach, promotion of physical, psychosocial, and spiritual health, training to have PC conversations, ACP.	Improve symptoms and QOL, PC education improves nurses’ knowledge, attitudes, and preparedness when caring for patients with cardiac disease in need of PC services
Vallabhajosyula, 2019^10^, USA, Retrospective cohort study	Evaluate the 15-year national utilization, trends, predictors, disparities, and outcomes of PC services use in cardiogenic shock complicating ACS	Higher comorbidity and acute organ failure. Higher in-hospital mortality	Social, emotional, and spiritual aspects of patients and families, multidisciplinary team	Lower hospitalization costs, shorter hospital length of stay, and lesser use of futile care. Greater use of DNR status and adopt life-limiting decisions and opt for non-hospitalization care at EOL.
Waller et al, 2013^33^, Australia, Brief methodological report	Explore the psychometric quality of a newly developed rapid screening measure to assess the supportive and palliative care needs of people with CHF.	Disability, physical, psychological, social, and informational unmet needs, symptom burden, comorbidities, greater physical symptom burden and social needs	Multidisciplinary care models that integrate a PC approach, QOL, NAT: PD-HF is the only tool to facilitate the concurrent assessment of unmet needs of HF patients, their caregivers, and their families, assessing patient symptoms, tailoring PC services to needs	PC services ensure that they are corresponding with patients’ needs, reduce the gap between the provision of specialist and community services, enabling HCPs to identify individuals who may require targeted early interventions, facilitate a conversation with the health care team, prompt further assessment and appropriate referrals to meet unmet needs

Notes: PC, Palliative care; ACP, Advanced care planning; QOL, quality of life; EOL, end of life; HCP, Health Care Professional; CHF, congestive heart failure; HF, heart failure; ESAS, Edmonton Symptom Assessment System; NAT: PD-HF The Needs Assessment Tool: Progressive Disease—Heart Failure; ACS, acute myocardial infarction; DNR, do not resuscitate.

### Definition of Palliative care

The World Health Organization’s definition of PC is widely used in the literature. It defines PC as “an approach that improves the quality of life (QOL) of patients and their families facing life-threatening illness, through the prevention, relief of suffering, early identification, comprehensive assessment, treatment of pain and other problems, physical, psychosocial, and spiritual”.^5^ “Palliative care refers to caring for patients and families facing life-limiting illnesses. It focuses on a holistic approach to treating the impact of illness and is often provided along with clinical care. It is patient-centered coordinated care that aims to relieve suffering and improve the QOL at all stages of the illness”.^34^

### The Use of Palliative Care Concept in Literature and Related Concepts

In the literature, PC in the elderly cardiac population is mainly discussed in association with heart failure management because many cardiovascular diseases, such as valvular heart disease and coronary heart disease in the elderly, lead to HF.^2^ Several articles have developed an understanding of PC by differentiating it from related concepts such as hospice care or end-of-life care. PC is administered to patients with a severe disease burden, starting early in the disease trajectory and continuing throughout the disease course. In contrast, hospice care provides care at the end of life.^11^ In addition, PC can be utilized along with curative treatment; however, hospice care involves only palliation of symptoms, without curative treatments.^10^ A referral to PC does not require a specific prognosis, while hospice care requires acceptance of death with a life expectancy of fewer than 6 months.^4,22^ Hospice care aims to improve the quality of the death and dying processes, whereas the purpose of PC is to relieve suffering and improve QOL.^6^

### Antecedents

**Figure 2.**
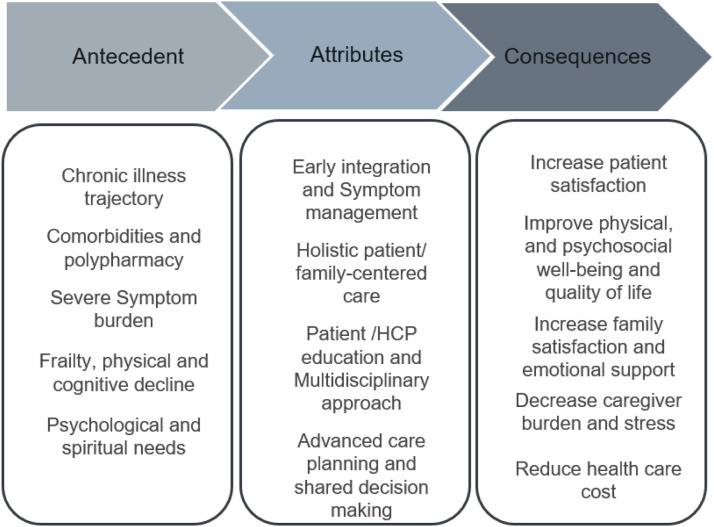
Antecedent, attributes, and consequences for palliative care for elderly with heart diseases.

Antecedents were events that existed before the concept was developed^14^ (Figure 2). In this review, the antecedents of palliative care for elderly patients with cardiac issues in tertiary settings are grouped into 5 major categories, which serve as the foundation of the concept.

#### Chronic Illness Trajectory

 HF has an unpredictable disease trajectory, with periods of stability interrupted by exacerbations, sometimes ending in sudden death.^6,11,12^ Approximately 40-50% of people with HF die within 5 years of the initial diagnosis.^29,31^ Therefore, patients with HF can benefit from PC throughout the disease course.

#### Comorbidities and Polypharmacy

Almost 58% of patients with HF have 5 or more comorbidities, including hypertension, diabetes, chronic kidney disease, and COPD.^17,26^ Multiple chronic conditions and fragmented care in the elderly result in polypharmacy, which leads to the complexity of treatment regimens and drug-related adverse effects, contributing to non-adherence.^17,20^ Thus, introducing palliative care for the elderly improves the self-management of comorbidities and treatment adherence.

#### Severe Symptoms Burden

Symptom burden negatively affects the QOL of the elderly. The most common symptoms include dyspnea, anxiety, depression, chest pain, nausea, pain, and delusions, which are associated with frequent hospitalization, mortality, and inadequate self-care. Almost 90% of older HF patients have reported experiencing dyspnea, and depression affects 13-77% elderly.^16^ These symptoms have a detrimental impact on the physical, psychological, and social well-being of elderly patients, significantly affecting their quality of life.^19^

#### Frailty, Physical and Cognitive Decline

Multimorbidity worsens physical and cognitive functions in older cardiac patients. Frail patients with HF are at higher risk of complications, prolonged recovery, readmission, disability, and mortality.^3,32^ Elderly patients with cognitive dysfunction can also experience poor self-care and inability to make decisions.^2,16,27^

#### Psychosocial and Spiritual Needs

Geriatric patients also suffer from psychosocial and spiritual issues characterized by hopelessness, isolation, emotional distress, a sense of uncertainty, loss of meaning in life, higher financial burden, loss of dignity, and increased dependence.^4,13,19^

### Attributes

Walker and Avant^14^ showed that attributes are characteristics that are frequently associated with the concept (Figure 2). The main attributes related to palliative care for older adults with cardiac conditions in tertiary care settings were categorized into four themes, which have been frequently discussed in the literature.

#### Early Integration and Symptom Management

PC is integrated early in the course of HF, where PC therapies can be used along with conventional therapies, and become the dominant treatment once the patient has progressed to advanced HF.^17,27,29^ In elderly patients with HF, symptom control and QOL should be prioritized to improve survival. Switching to PC does not imply discontinuing medical therapy, because it lowers the risk of symptomatic deterioration.^3,12,32^

#### Holistic patient-and Family Centered Care

In elderly cardiac patients entails treating the patient as a whole, focusing on general health outcomes rather than disease-specific outcomes, and addressing the patient’s and family's emotional, spiritual, and psychological needs.^17,19,27,29^ PC is a patient-family-centered approach that improves overall health based on values, goals, and preferences, regardless of prognosis.^6,11^

#### Patient/HCP Education and a Multidisciplinary Approach

In several studies, HCP, patient, and family PC education were effective in integrating PC while promoting patient self-efficacy and participation in decision-making.^11,13,16^ Most patients and families desire open, honest information about the disease, which requires effective communication and collaboration between healthcare providers and patients.^6^

#### Advance Care Planning and Shared Decision-Making

Advance Care Planning (ACP) is a process by which patients plan for future health if their health declines, which assigns the surrogate decision-maker and ensures that medical care is tailored to individual values, goals, and preferences. Shared decision making is crucial for exploring patients’ wishes regarding ACP.^4,7,20,29,30^ Many elderly individuals have implantable devices, such as pacemakers, defibrillators, and ventricular assist devices. Discussions, communication, and decision-making before device implantation and modification should be integrated into the ACP.^6,20^

### Model Case

The model case illustrates the concept in pure form.^14^ This model case is an example that demonstrates all the attributes of palliative care required for the elderly with heart disease. A 60-year-old man was diagnosed with heart failure (HF). A cardiac resynchronization therapy defibrillator (CRT-D) was introduced along with optimal pharmacological therapy after a detailed discussion regarding patient goals, treatment options, and device deactivation at the end of life. A multidisciplinary team was involved, and a PC advanced practice nurse (APN) provided supportive care. The APN educated the patient and his wife about HF management, performed ongoing follow-ups, referred him to a social worker for financial concerns and cardiac rehabilitation, and completed the ACP. At the age of 65, he was admitted for HF exacerbation, and multidisciplinary interventions were initiated. Transcatheter mitral valve repair was recommended at that time. The risks and benefits of the invasive procedure, goal of care (GOC), and treatment options were discussed, but the patient declined this treatment. He already had an ACP document that was modified based on the current discussions. Milrinone and furosemide infusions were initiated to manage the symptoms; however, his respiratory symptoms and pain did not improve. The PC team assessed the patients’ needs and planned the interventions accordingly. Oral extended-release hydromorphone 6 mg q12 h and PRN hydromorphone were ordered along with HF symptom control therapy, which improved his symptoms and ability to perform daily activities. His vital signs were stable, and no adverse effects were observed. Post-discharge, treatment with HF medications and hydromorphone was continued, and APN managed the follow-ups. Owing to the ongoing psychological, social, and spiritual support provided by the PC services, he was able to manage his disease without frequent hospital visits. He was optimistic, and independent, and spent time with his family.

In this case, the PC approach was introduced early at the time of heart failure diagnosis, which was helpful because of its unpredictable trajectory. Along with curative treatment to manage symptoms, the goals of care were discussed, including device deactivation at the time of CRT-D insertion. The multidisciplinary team, including the cardiology team, palliative/supportive team, and APN, was involved in patient care and shared decision-making was encouraged. The APN followed up with the patient, provided education to the patient, and his wife and completed the ACP. Holistic patient-centered care is provided by assessing the patient's needs (physical and financial) and referring to appropriate resources such as social workers and cardiac rehabilitation. When the patient was admitted with progression of heart failure and a procedure was recommended, the goal of care discussions was completed, and the patient chose medical management with shared decision making. Milrinone and Lasix infusions were ineffective in managing dyspnea and pain. The PC team was actively involved, prescribed opioids for dyspnea and pain control, assessed patient needs, and modified the ACP document according to patient values and preferences. It is best practice to review ACP and GOC for each admission, when there is a change in the health status of the patient and before any procedure. Because of the early integration of PC with all the required attributes, the patient and his wife were satisfied with the care and received timely psychological, social, and spiritual support as a result of the holistic patient/family-centered care approach. The patient had a good quality of life and unnecessary hospital admissions were avoided.

### Borderline Case

A borderline case has most of the concept’s defining attributes, but not all of them.^14^ This case highlights the provision of appropriate PC to the elderly with cardiac issues in hospitals, yet it falls short of providing the optimal level of care as mandated by all attributes. A 65-year-old male with a history of coronary artery bypass was previously admitted three times for HF exacerbation. Each time symptoms were managed, and the patient was discharged. The patient lived with a spouse dependent on him for care and always worried about her. There was no discussion about prognosis and ACP, and a multidisciplinary team was not involved early in the HF trajectory. At the age of 68 years, the patient was admitted with advanced HF with fluid overload (unresponsive to intravenous or oral diuretic), and guideline-based medical therapy could not be continued because of low blood pressure (80/54 mmHg). The patient had a limited functional capacity, breathlessness, and depression. The multidisciplinary healthcare team explained the treatment options to the patients and his son and wife and discussed the GOC at a family meeting. The patient refused aggressive treatment in favor of conservative treatment. ACP was completed and the GOC discussion was documented. The cardiologist discussed the patient's poor prognosis and short life expectancy and the PC team was consulted for supportive and transitional care. Medication optimization, patient and caregiver education through palliative care APN, and incorporation of cardiac rehabilitation improved the patient's condition, and he was transferred to hospice care. Eventually, his urine output decreased, and his respiration deteriorated. He and his family opted for no resuscitation during the cardiac arrest. Therefore, intravenous morphine infusion was initiated for pain relief in consultation with the PC specialists. The patient and family were satisfied with the care that the patient received in the last 6 months of his life. However, earlier PC support could have helped the patient cope with personal issues and better manage his diseases.

In this case, the PC team was consulted when the patient’s condition deteriorated and progressed to advanced HF. All the main attributes of PC were implemented, such as symptom management; multidisciplinary approach; GOC and ACP discussion and documentation; provision of holistic patient family centered care by addressing patient needs, values, and wishes; patient family education; and shared decision-making based on discussions in family meetings. However, PC was not incorporated early despite multiple hospital admissions, rapid progression of HF (unpredictable trajectory), and patients’ need for self-care, social support (caregiver role), and psychological support (depression). The timely incorporation of PC could have improved patient satisfaction and quality of life while addressing patient and family needs efficiently and utilizing health care resources appropriately.

### Contrary case

The Contrary cases are examples that do not relate to Concept^14^. The case scenario showed that best practices related to palliative care were not implemented when caring for the elderly in tertiary care. A 60-year-old single female with comorbidities, post-6-year myocardial infarction, and left-sided HF (EF = 30%). She was hospitalized 5 times. Her family doctor visits have been rushed, she has not received interdisciplinary care, and PC has not been offered. She was admitted to the hospital with HF exacerbation (EF = 20%), acute renal failure, and functional decline. During the rounds, the doctor spoke to her briefly and said, “We’ve come to the end, and you may only have a few months left." Cardiology, PC, and hospice teams were not consulted. There were no discussions regarding ACP or EOL support options, wishes, or resuscitation preferences. The patient was discharged without any transitional care. She was given pamphlets on PC and hospice services, but no referrals were made for HF follow-up. She lives alone and has not discussed the seriousness of her condition with her daughters because she does not want them to worry about her. She feels hopeless and anticipates her death. Her friends constantly hear her say, “I'm just tired and want all these to end.”

Despite several indications, including multiple admissions, advanced HF with an EF of 20%, comorbidities, functional decline, and need for social support, palliative care was not provided to the women throughout the course of the disease process. Although the patient was provided with educational material, all attributes of palliative care for elderly patients with heart disease were absent.

### Consequences

In the Walker and Avant approach, consequences were defined as the outcomes of the concept.^14^ The integration of PC for older adults in tertiary care settings has positive outcomes at the individual, family, and healthcare system levels. Early PC in the older cardiac population enhances patient satisfaction and facilitates understanding of the diagnosis. In addition, it improves the patient’s physical and psychosocial well-being and QOL by increasing symptom control, self-care, and treatment adherence; reducing financial burden and stress; and encouraging avoidance of futile invasive interventions.^3,22,29,32^ The involvement of caregivers in providing PC to the elderly is crucial for increasing their satisfaction and reducing stress, improving patient self-care and treatment adherence, and improving emotional, spiritual, and bereavement support for the family.^16,17,22^ Early hospital-based PC improves QOL and symptoms of HF in older patients and is associated with decreased healthcare costs related to lower hospital readmissions, decreased length of hospital stay, and fewer invasive procedures^16,22,29^

### Empirical Referents

Empirical referents are real-world instances that determine the existence of a concept.^14^ Most validated tools, such as the Supportive and PC Indicators Tool (SPICT), surprise questions, and the gold standard, aim to identify patients who should receive PC and those at risk for deterioration or death.^28,30^ In contrast, the Needs Assessment Tool: Progressive Disease—Heart Failure (NAT: PD-HF) is a validated tool used by the HCP to evaluate the needs of patients with cardiac disease and is consistent with the current perspective of PC.^2^

## Discussion

The world’s aging population is estimated to grow more than threefold by 2050, and the prevalence of HF is increasing with this aging population.^4^ HF is the leading cause of hospital admissions owing to its unpredictable nature, and geriatric patients are at a higher risk of sudden death and experience economic costs of treatment and emotional suffering.^8,25^ Despite this, patients with cardiovascular disease are less likely to receive high-quality PC than those with other chronic diseases such as cancer.^9^ PC for patients with heart failure (HF) is often delayed due to prognostic uncertainty, lack of training among healthcare providers related to PC principles, lack of shared decision-making and ACP, and delayed referral until all HF therapies have been exhausted.^8^

International cardiology organizations advocate the early introduction of PC into the HF disease trajectory.^2,24,35^ The purpose of providing PC is to ensure the highest standard of quality of life for the patient and to implement interventions at all stages of heart failure. This requires, a multidisciplinary approach that focuses on communication, shared decision-making, advance care planning, symptom control, psychological and spiritual care, and offers support to families and caregivers to cope with the patient’s disease process and beyond, including care after death.^7,17^ Misconceptions and lack of knowledge regarding PC among patients and HCPs are barriers to quality and timely care.^11^ The persistent stigma surrounding the term “palliative care,” which is often associated with death, dying, and end-of-life care, presents a significant obstacle to the integration of PC.^7^ Studies have found that patients overestimate their life expectancy by 40%, and many providers and patients believe that PC should be used only at the end of life.^6,23^ Mandatory HCP education on palliative care concepts, symptom management, and communication skills as well as institutional policies can ensure the early incorporation of PC.^7,11,26^ Integrating early PC in geriatric patients does not automatically imply the discontinuation of medical treatment. In contrast, guideline-based medical therapy helps to maintain ventricular function, renal function, and blood pressure targets, reducing dyspnea, arrhythmias, and the risk of symptomatic deterioration.^3^

Frailty is 6 times more likely to be observed in patients with HF because of the significant burden of comorbidities, polytherapy, and physical and cognitive decline. Anxiety, depression, cognitive impairment, and dementia are common among elderly patients with HF and are associated with poor clinical outcomes.^3^ Severe dyspnea can often be effectively managed using vasodilators and diuretics, as tolerated, and the treatment of anxiety and depression in patients with advanced disease may benefit from the use of selective serotonin reuptake inhibitors (SSRIs) and spiritual support for both patients and their families.^17^ Deprescribing improves the clinical care and QOL of older adults, and hospitalization provides an excellent opportunity to review a patient’s pharmacological regimen and stop unnecessary medications.^3^ A multidisciplinary approach is required to provide comprehensive PC at all stages of heart failure. A multidisciplinary team comprising the primary physician, nursing staff, pastoral care/bereavement support, and other support staff is essential.^17^ Interdisciplinary teams should communicate openly with family members, patients, and other team members.^7,17,22^ One study showed that over 80% of hospitalized older patients with HF did not have documented advance directives in their medical records at any point during the 5-year study period.^18^ To ensure patient-centered care, starting PC conversations, discussing options, and establishing goals of care are important. The ACP process requires effective communication and shared decision-making. It is essential to hold the initial discussion in a non-clinical setting when the patient is stable and able to participate, as well as after an unexpected hospital admission, to reflect on the current plan, and when the patient has recovered.^20^ Decisions around ICD deactivation can be more complex than its implantation, and providers rarely discuss ICD deactivation, resulting in ICDs remaining active until death.^4,6^ Deactivating an ICD does not result in immediate death, as it does with an LVAD. Patients' beliefs and wishes regarding end-of-life care should guide device implantation and deactivation decisions.^6^ A study was conducted to investigate the unique PC needs of elderly patients with advanced HF and to uncover the factors that influence quality of life, including existential or spiritual well-being, physical well-being, psychological well-being, and educational level. The findings demonstrated that these factors are significantly linked to the quality of life of elderly HF patients and emphasized the importance of holistic care in addressing these PC needs.^19^

Early PC prevents unnecessary and harmful diagnostics and treatments, reduces symptom burden, reduces hospital admissions and the cost of health care, and improves patients and caregivers’ quality of life by addressing their needs and wishes.^3,7,8,18^ SPICT and other PC tools have been designed to identify older hospitalized patients at risk of dying within 1 year and who might benefit from PC^30^ However, organizational guidelines recommend providing PC to patients with HF based on the assessment of symptoms and needs rather than individual estimates of remaining life expectancy.^35^ The NAT: PD-HF is the most appropriate validated tool to evaluate patient and caregiver needs that assists in early PC Integration and corresponds to recent guidelines.^2^

### Implications for Advanced Nursing Practice

APN expertise in research, education, clinical practice, consultations, collaborations, and leadership in providing PC improves the QOL and satisfaction of elderly patients and caregivers. APNs evaluated the knowledge gap in PC philosophy, pharmacology, and symptom management in tertiary care, and identified the need for continuing education.^13^ APNs are vital for providing supportive care to elderly patients with HF through educating nurses and patients. An educational project implemented by APNs in a hospital improved perceived knowledge and skills related to PC conversations.^11^

An APN assesses patient needs in direct patient care, facilitates communication, promotes self-care, supports care goals, and facilitates collaboration among healthcare providers.^29,33^ APNs incorporated ENABLE (Educate, Nurture, Advise Before Life Ends), a PC model that identifies patients’ and caregivers’ needs and provides education and support to address those needs.^21^

Providing quality  PC services for the elderly requires APNs' consultations and collaboration skills. In a pilot project, HF patients identified nurse practitioner consultations as a key benefit of the program for assessing PC needs and collaborating with professionals in hospital and community settings on their behalf.^7^ APNs are innovative leaders and researchers who implement evidence-based strategies to improve PC for the elderly, including PC models, integrated care pathways, advanced care planning, and interdisciplinary workshops.^29^

### Strengths and Limitations

The strengths of this analysis include a thorough search of relevant literature, the use of Covidence software for comprehensive data management and analysis of studies, and adherence to the inclusion and exclusion criteria. Palliative care was discussed in terms of its specific context and population, which included older cardiac patients in tertiary care settings. This provides a deeper understanding of the concept, which can be translated to similar populations and clinical settings. Among the limitations of the analysis, studies on illnesses other than heart disease were excluded, which may have led to valuable inferences. In addition, the findings of this conceptual analysis may not be applicable to diverse clinical contexts and populations.

## Conclusion

Given the growing complex needs of geriatric patients with heart disease, the health system should emphasize the early integration of PC services in tertiary care. The lack of continuity of PC could be improved by connecting tertiary care services to community services through technology, developing a transitional care program to ensure proper follow-up after hospital discharge, and delivering necessary supportive care to elderly cardiac patients. From a research perspective, there is a significant opportunity to fill this gap by exploring ways to identify the PC needs of older patients with HF and to provide them with timely PC. Further research is needed to evaluate patients’ experiences with PC, and whether PC services meet these needs.
